# A retrospective study to assess the evaluation of living related kidney donors and their outcomes following nephrectomy at Kenyatta National Hospital

**DOI:** 10.1186/s12882-017-0585-7

**Published:** 2017-05-25

**Authors:** A. Muturi, V. Kotecha, S. Kanyi

**Affiliations:** 1 University of Nairobi, Nairobi, Kenya; 2 Department of Surgery Kenyatta National Hospital, Nairobi, Kenya

## Abstract

**Background:**

Kidney transplantation is the renal replacement therapy of choice for end stage renal disease. To ensure safety regular audit of the donation process is necessary. The aim of this study was to assess the evaluation of potential living related kidney donors and document their outcomes following nephrectomy.

**Methods:**

This was a retrospective descriptive study involving all living related kidney donors seen at Kenyatta National Hospital (KNH) renal unit from 2010 to 2014.

Upon approval by KNH/ERC, the records of all kidney donors were retrieved. Demographic characteristics, number of potential and actual donors, their clinical, laboratory and radiological data as well as documented complications and deaths were recorded. SPSS version 17(Chicago, Ilinois) was used for data entry and analysis. Chi square test and Mann Whitney U test were used as tests of association for categorical and continuous data respectively, with *P* value set at <0.05.

**Results:**

Median age of the donors was 34 years (IQR 31–39). First-degree relatives were majority(84.5%). Renal function assessment was done using mean glomerular filtration rate (GFR) from the radionuclide scan (DTPA) and serum creatinine levels. The donors had a mean GFR of 99.2 ± SD 6.6. All the haematological and biochemical tests were within normal. Majority(42.9%) were HLA compatible, but data on HLA typing was missing for 22% of the patients records. On CT angiogram, single renal artery and single renal vein were found in 94 and 88% respectively. Immediate complications included excessive bleeding(2%) and breach of other cavities (4%). Paralytic ileus (32%) and atelectasis (27%) were the most common early postoperative complications. There was no mortality.

**Conclusion:**

Our study reports no fatality but significant post-operative complications. These are significant findings that may be used to review and improve care and to educate potential kidney donors on the safety of this procedure in our centre, in a bid to widen the pool of potential living kidney donors.

## Background

Globally, the incidence of end-stage renal disease (ESRD) is consistently increasing, currently standing at 6% per year [1]. As of 2006, Kenya’s national prevalence of ESRD stood at 15.6 per million population (PMP) with a reported 12.7% rise in prevalence between the year 2000 and 2004 [2]. Chronic kidney disease (CKD) is at least three to four times more frequent in Africa than in developed countries with dialysis treatment rate ranging from 70 PMP in South Africa to 20 PMP in the most of sub-Saharan Africa, and transplant rates averaging 4 PMP [3].

In Sub Saharan Africa, patients with ESRD are relatively young, ranging between 20 and 50 years compared to the developed world who are elderly [4, 5]. It is the productive age group that is affected, and that has a negative economic impact. The management of ESRD by dialysis is costly, especially in the developing world where individual patients are directly financially responsible for their care [6].

Kidney transplantation therefore is the best form of management because it not only corrects renal functional impairment, it also provides normal or near normal quality of life for recipients and is the most cost-effective therapy in the long run [6, 7]. In most parts of Africa, maintenance haemodialysis is beyond the reach of most people due to cost and shortage of dialysis centres [7]. This is compounded by lack of living donors, laws that do not allow for cadaveric donors, few transplant surgeons and prohibitive costs of anti-graft rejection drugs [8–10].

Kidney donation is free and voluntary since commercial organ donation is considered unethical and is prohibited in most parts of the world [11]. In Kenya the transplant program is relatively new facing numerous challenges ranging from shortage of transplant surgeons, reliance on living related donors, cultural barriers as regards organ donation and the cost of transplant surgery that is out of reach for most people. Majority are related donors and while consent process tries to ensure a donor is well informed about the risks, every attempt is made to dispel myths and strongly held false beliefs to ensure the decision is voluntary. This is done to eliminate cases of potential donor going through the process under duress. The donor should be assured that by donating a kidney they face no major risk to their life at present or in the future [12]. Regular audits are therefore necessary to ascertain complications rates and for purposes of quality assurance [13].

The guidelines used at our renal unit for assessment and follow-up of donors are derived from the European guidelines [12, 14].

It is with this background that we undertook this study to document the demographic characteristics, the pre-operative evaluation and the complications of living related donor nephrectomy at KNH.

## Methods

This was a retrospective descriptive study involving all living donors seen from 2010 to 2014.

The study was carried out at the renal unit of Kenyatta National Hospital; the largest referral and teaching hospital in Kenya with a bed capacity of 1800.

The renal unit offers specialized care such as dialysis and renal transplant, having the highest rate of performing renal transplants within East Africa.

Records of all potential living kidney donors and from that, the actual living donors were retrieved. Incomplete records of potential and actual kidney donors were excluded.

Demographic characteristics, number of potential and actual donors, their clinical, laboratory and radiological data as well as documented complications and deaths were recorded.

### Data analysis

Statistical package for social sciences (SPSS) version 17 (Chicago, IL) was used for data analysis. Continuous variables such as age, laboratory parameters and radiological and intra-operative measurements of renal arteries branching distance from the aorta. Categorical variables included gender, biologic relationship of the donor to the recipient and renal anomalies noted.

Means and medians were used to describe the data. As measures of association, MannWhitney ‘U’test and Chi square were used for continuous and categorical variables respectively. Statistical significance was considered for *P* value of <0.05.

## Results

**Fig. 1:**
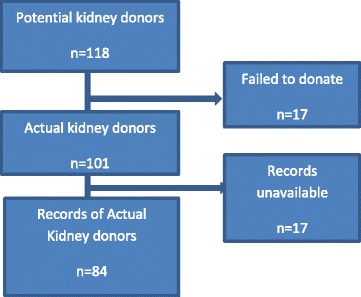
Kidney donor records. There were 118 potential donors, all found suitable by screening, but 17 of them didn’t donate for various reasons including: declining at the last minute, ABO incompatibility, active tuberculosis and recipient’s death

During the study period 118 were screened as potential donors but only 101 kidney donations took place. We retrieved records for 84 donors (Fig. 1). Majority of the donors were male 61.9%. The median age of the actual kidney donors was 34 (IQR 31–39). The reasons why some potential donors didn’t donate included: potential donor declining, recipient death among others (Table 1).

**Table 1:** Donor Sociodemographic characteristics

Variable	Frequency (%)
Kidney donor	
Sex	
Male	52 (61.9)
Female	32 (38.1)
Age	
Mean (SD)	34.8 (6.8)
Median (IQR)	34.0 (30.0–39.0)
Min-Max	22.0–49.0
Mode	36.0
Relationship with recipient	
Aunty	1 (1.2)
Brother	36 (42.9)
Cousin	7 (8.3)
Daughter	1 (1.2)
Father	6 (7.1)
Husband	1 (1.2)
Mother	1 (1.2)
Nephew	2 (2.4)
Niece	1 (1.2)
Sister	25 (29.8)
Son	2 (2.4)
Uncle	1 (1.2)
Reasons for not donating	
Declining	11
Recipient death	4
Active tuberculosis	1
ABO incompatibilty	1
Takes alcohol	
Yes	32 (38)
No	52 (62)
Smoking	
Yes	15 (18)
No	69 (82)

Majority of the donors were male (61.9%),most of them siblings to the recipient, with a median age of 34.8 years and only about 18% with a smoking history

All the kidney donors underwent biochemical, microbiological and haematological tests, all of which were normal (Table 2).

**Table 2:** Haematological, microbiological and biochemical tests before surgery

Parameter	Mean (SD)	Normal range
Renal assessment		
GFR	99.2 (6.6)	>90 mls/min/1.73m^2^
Urea	4.8 (1.7)	2.5–7.1 mmol/l
Creatinine	104.0 (19.9)	60–125 umol/l
Electrolytes		
Na^+^	139.4 (6.1)	135–145 mEq/L
K^+^	4.3 (0.5)	3.5–5.5 mEq/L
Cl^−^	101.0 (1.4)	95–105 mEq/L
Ca2^+^	2.4 (0.9)	4.5–5.5 mEq/L
Po4^−^	1.4 (1.1)	2.5–4.5 mg/dL
Lipid profile		
HDL	1.8 (0.2)	>1.17 mmol/L
LDL	2.3 (0.8)	<2.3 mmol/L
TG	1.6 (0.6)	<1.7 mmol/L
Total cholesterol	3.7 (0.7)	<5 mmol/L
Liver function test		
AST	33.2 (6.3)	0–42 u/L
ALT	24.4 (8.2)	0–42 u/L
ALP	48.2 (22.5)	25–100 u/L
GGT	48.3 (15.7)	8–65 u/L
Total bilirubin	15.1 (6.9)	5–17 umol/L
Direct bilirubin	6.1 (2.3)	0–7 umol/L
Indirect bilirubin	5.6 (0.8)	1–17 umol/L
Albumin	38.6 (3.9)	35–55 g/L
Total protein	67.3 (7.3)	67–78 g/L
Fasting glucose	4.7 (0.4)	3.8–6.1 mmol/L
Haematological profile		
WBC	6.9 (2.1)	4.5–11 × 10^9^/L
Hb	13.2 (1.2)	12–17.5 g/dL
Platelet count	289.9 (85.2)	150–400 × 109/L
Coagulation profile		
Aptt	30.0 (2.0)	25–40 s
INR	1.1 (0.1)	0.8–1.2

All the tests were within normal range. Renal function, liver function, coagulation profile, lipid profile, coagulation profile, compatibility testing and infectious disease screen were done. Infectious disease screen included: HIV, hepatitis B and C, CMV, VDRL and malaria test

During the follow up period haematological and biochemical laboratory tests were done, 1 month from the day of discharge from the hospital. The renal function tests were normal but there was a negligible drop in GFR. The rest of the tests were normal.

In terms of tissue compatibility, all were ABO compatible, 42.9% were HLA compatible, 34.5% were not compatible and for 22.6% of the donors, this information was missing from their files.

**Fig. 2:**
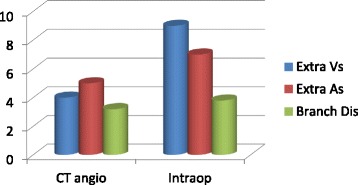
Relating the CT angiogram findings and intra-operative report on vascular anatomy. The intra-operative findings of the donor kidney was largely consistent with the CT scan findings but with slight variation when vascular anatomy is considered. While the branching distance from the aorta was as predicted by the CT angiogram, the imaging underestimated presence and number of extra vessels

The kidney donors underwent preoperative kidney ureter and bladder (KUB) ultrasound, abdominal CT-scan and CT-angiograms to detect the number of kidneys and any abnormalities in them. There was one patient who had a simple cortical cyst but otherwise a well-functioning left kidney. Majority (94%) of the donors had a single renal artery. Two thirds of the donors had 3 branches from the main renal artery as it entered the kidney. The renal artery had a good length from its origin from the aorta with a mean length of 3.2 cms. The renal vein was single in most of the donors (88%) (Table 3 and Fig. 2).

**Table 3:** Radiological assessment

Number of kidneys	
Two kidneys	84 (100%)
Abnormal kidney	1 (1.2%)
Simple cortical cyst	
Number of renal arteries	
1.00	79 (94.0%)
2.00	3 (3.6%)
3.00	1 (1.2%)
Information Missing	1 (1.2%)
Renal artery Branches	
1.00	3 (3.6%)
2.00	4 (4.8%)
3.00	65 (77.4%)
4.00	2 (2.4%)
Missing	10 (11.9%)
Branching distance from Aorta in Cms, mean (SD)	3.2 (0.6)
Number of renal Veins	
1.00	74 (88.1%)
2.00	1 (1.2%)
Accessory renal veins	4 (4.8%)
Information Missing	5 (6%)

Radiological screening was by use of KUB ultrasound with only one simple renal cortical cyst found. The next modality was CT angiogram to define the renal anatomy, position, size and vascular anatomy. Majority of the donors had a single renal artery 94% with two thirds of them having 3 branches from the main renal artery as it entered the kidney. The renal artery had a good length from its origin from the aorta having a mean length of 3.2 cms. The renal vein was single in most of the donors 74%

Complications were categorised as immediate, early and late. Although there were a few complications, there was no mortality. Immediate complications were bleeding (2%) and breaching of other cavities like the pleural cavity(3.6%) and peritoneal cavity(1%). Postoperative ileus was the most common early complication (32%) and with second most common complication being atelectasis (21%). Persistent pain was the most common late postoperative complication (68%) (Table 4).

**Table 4:** Complications following nephrectomy

Variable	Frequency (%)
Mortality	0
Immediate Complications	
Excessive bleeding	2 (2.4)
Bowel injury	0
Vascular injury	0
Solid visceral organ injury	0
Anaesthetic	0
Other complications	
Breach of pleural space	3 (3.6)
Breach peritoneum	1 (1.2)
Post-operative complications Early	
Bleeding	2 (2.4)
Ileus	27 (32.1)
Wound infection	5 (6.0)
UTI	2 (2.4)
Pneumonia	2 (2.4)
Atelectasis	18 (21.4)
DVT	0
Post-operative complications Late	
Persistent pain	57 (67.9)
Hernia	0
Paraesthesia/numbness	13 (15.5)

The commonest early postoperative complication was ileus followed by atelectasis. There was no mortality in this cohort. Late complications were minimal but included paraesthesiae and persistent pain

There was a weak association between male gender and risk of developing post nephrectomy complications. The other patients’ characteristics considered were not significantly associated with risk of developing complications (Table 5).

**Table 5:** Factors affecting complications

Variable	Complications	No complications	OR (95% CI)	*P* value
Age	34.8 (6.9)	35.3 (6.9)	-	0.774
Sex				
Male	46 (88.5%)	6 (11.5%)	3.0 (1.0–9.5)	0.054
Female	23 (71.9%)	9 (28.1%)	1.0	0.081
Takes alcohol				
Yes	26 (81.3%)	6 (18.8%)	0.9 (0.3–2.8)	0.867
No	43 (82.7%)	9 (17.3%)	1.0	
Smoking				0.062
Yes	15 (100.0%)	0 (0.0%)	-	
No	54 (78.3%)	15 (21.7%)		
BMI	26.8 (3.8)	26.5 (2.9)	-	0.761

Several patient’s characteristics were assessed for association with the odds of developing complications. Only male gender was significantly associated with risk of complications

During follow the up period, the donors had assessment of their clinical and laboratory parameters pertaining to general wellbeing and renal function among others. They were all within normal ranges (Table 6).

**Table 6:** Clinical and laboratory evaluation during follow up-1 month after discharge

Variable	Mean (SD)	Median (IQR)	Min-Max
Pulse	72.8 (8.4)	70.0 (68.0–76.0)	60.0–101.0
Respiratory rate	22.3 (5.6)	22.0 (20.0–23.0)	18.0–70.0
Weight	65.5 (10.6)	65.5 (58.0–65.0)	58.0–73.0
Estimation of GFR	93.7 (8.1)	90.6 (89.6–99.3)	76.0–140.0
Laboratory			
Urea	6.2 (8.2)	4.7 (3.9–6.3)	2.1–64.0
Creatinine	106.4 (17.0)	106.0 (90.0–124.0)	66.0–130.0
Na+	137.4 (3.5)	136.0 (136.0–139.0)	128.0–149.0
K+	4.2 (0.4)	4.1 (3.9–4.6)	3.4–5.1
Ca+	2.3 (0.2)	2.4 (2.3–2.5)	1.5–2.6
Po4	1.3 (0.3)	1.3 (1.1–1.5)	0.9–1.9
AST	36.0 (6.2)	36.0 (32.0–41.0)	15.0–47.0
ALT	26.7 (8.0)	26.0 (21.0–32.0)	9.0–47.0
ALP	46.5 (18.9)	45.5 (35.5–54.0)	13.0–99.0
GGT	48.3 (15.3)	46.0 (40.0–60.0)	21.0–120.0
Total biliburin	14.7 (6.3)	13.9 (12.1–15.8)	9.6–54.0
Direct bilirubin	5.89 (2.2)	6.0 (4.6–6.4)	2.1–17.1
Albumin	38.4 (3.2)	38.0 (36.0–40.0)	34.0–50.0
Total protein	69.0 (7.4)	69.0 (64.0–74.0)	50.0–84.0
WBC	7.2 (2.2)	6.9 (5.1–8.9)	4.1–13.6
Hb	12.1 (1.3)	12.1 (11.2–12.9)	9.7–15.3
Platelet count	312.9 (73.7)	307.0 (264.0–384.0)	182.0–456.0

One month after discharge following the donor nephrectomy, the patients were seen at the nephrology clinic where their clinical and laboratory parameters pertaining to renal function and general wellbeing were performed. They were largely normal indicating a functioning level that was not affected by the nephrectomy at least as per that point of follow up

## Discussion

Majority of people who donated kidneys were young adults with a median age of 34 years. This finding differs from a study by Najarian from Minnesota whose donors were almost twice the age with a mean age of 61 years, but with similar post nephrectomy complication rates as our study [15]. Almost 60% of the donors were males. Younger persons can tolerate the trauma of surgery better and also have less comorbidity making them more suitable kidney donors. Like the recipients, in our set up the donors are young and in their most productive time of their life and it is therefore crucial for the operation to be safe.

In our centre majority of the donors were first-degree relatives 84.5% followed by second-degree relatives 14.3%, with a case of a man donating a kidney to his wife. In Kenya we don’t have cadaveric organ donation, we rely on living related donors.

Biochemical haematological and radiological tests were performed prior to kidney donation to ascertain eligibility and suitability for kidney donation. In this study the mean GFR was 99.2 mls/min/m^2^ prior to surgery and 97.7mls.min/m^2^ after nephrectomy. A CT- KUB and CT angiogram were done to discern the renal anatomy and presence of two normally functioning kidneys and rule out any disease like stone and parenchymal pathologies. In the present study all kidneys were normal except one who had a renal cortical cyst. The CT-angiogram and KUB provides a preoperative map to the surgeon on what to expect in a bid to make the surgery safer [16]. In the present study majority of the donors had a single renal artery (94%), and single renal vein (88%). Anatomists have studied the variance in renal vascular anatomy amongst Kenyans and several variations have been identified with 14.3% having an additional renal artery [17]. In the present study 4.7% had additional renal arteries. Similar study in donor nephrectomy patients revealed additional renal arteries in 27.7% [18]. Although presence of anomalous and supernumerary vessels is not a contraindication to donation, it is crucial to be aware of accessory renal vessels to avoid inadvertent injury which can lead to massive blood loss [17, 18]. The renal artery length was also studied on the CTA, with the donors in the present study having a mean renal artery length of 3.2 cm. It is important to have adequate length to enable successful tension free revascularisation. Information pertaining to the length is vital to the surgeon’s plan on the method of revascularisation of the kidney [18]. Some centres prefer renal artery anastomosis to the common iliac artery which has higher flow and is slightly larger hence easier to handle, but when short renal artery is encountered one may plan for anastomosis to the internal iliac artery [19].

Donor related complications in our setting were few. Immediate complications were; excessive intraoperative bleeding (2%) and pneumothorax (4%). From the present study, pneumothorax is the most common immediate complication which compares well with other studies [14]. Bleeding is more common after laparoscopic kidney donor nephrectomy, but at KNH we harvest the kidney by open access [20]. We had no patient who required re exploration. The complications need to be quickly recognised to avoid fatalities.

Other less severe early complications were postoperative ileus (32%) and atelectasis (21%). Postoperative ileus is not life threatening although it may prolong hospital stay and add some discomfort and anxiety to the donor. Atelectasis is the commonest complication after a laparotomy [20], in our study the loin incision was used which also can lead to atelectasis if the patient also has poor pain control leading to minimal chest excursion [20].

Persistent pain was the most common late postoperative complication (68%). Sixteen percent of the donors also reported numbness over the wound and in the loin. Numbness is a result of severing the sensory subcostal nerves and traction on the femoral nerve [20].

Cigarette smoking and alcohol consumption are not a contraindication to kidney donation but they have been associated with higher odds of developing complications after nephrectomy. This study looked at the risk factors for potential complications and only male gender had statistically significant correlation with complications. Other reported risk factors for potential donor complications include: smoking, age above 50 years, hypertension and weight equal to or greater than 100 kilogrammes [21].

### Interpretation

The main findings the study are similar to kidney donor studies done elsewhere and reinforces the need for regular audits to guarantee safety. Being a small retrospective study with a limited followup period poses a challenge in making robust conclusions and recommendations but overall, for our relatively new transplant center coupled with the challenges we encounter, will inform change in some aspects of intra-operative and postoperative care to have better outcomes.

### Limitations

This was a retrospective study relying on records and therefore there were cases of incomplete data entry and missing records. Secondly,the follow-up period for the donors was short, ranging from 2 weeks to a month. This means we do not have information on long term overall wellbeing and quality of life of the donors after nephrectomy.

## Conclusion

Kidney transplant is the ideal form of renal replacement therapy. In Kenya the source of the organ is living related donors, and people donate out of the need to help their kin live a near normal life and reduce cost associated with treating renal failure. To make the operation safe and successful, a number of blood tests, radiologic tests, and tissue typing are necessary.

Our study reports no fatality but significant early and late postoperative morbidity. This point to the need to institute measures both during nephrectomy and in the postoperative care to minimise these complications. Though there are significant complications, none was fatal and thus the data can be used to educate potential kidney donors on the overall safety of this procedure in our centre in a bid to increase the donor pool.

## Abbreviations

ABPM: Ambulatory blood pressure measurement
AKD: Actual kidney donor
APTT: Activated partial thromboplastin time
BMI: Body mass index
BP: Blood pressure
CKD: Chronic kidney disease
CT: Computer tomography
DTPA: Diethylenetriaminepentaacetic acid
DVT: Deep venous thrombosis
ECG: Eectrocardiogram
ESRD: End stage renal disease
GFR: Glomerular filtration rate
HIV: Human immunodeficiency virus
HSV: Herpes simplex virus
HTLV: Human t cell leukemia virus
KNH: Kenyatta National Hospital
KUB: Kidney ureter bladder
PE: Pulmonary embolism
PKD: Potential kidney donor
PMP: Persons per million population
PSA: Prostate specific antigen
PT: Prothrombin time
SPSS: Statistical package for social sciences
